# Inflammation as It Occurs in the Different Tissues of the Eye; Being a Complement to the Author's Reports on Inflammation and the Healing Process

**Published:** 1845-07

**Authors:** T. Wharton Jones

**Affiliations:** Lecturer on Anatomy, Physiology, and Pathology, at the Charing Cross Hospital; Corresponding Member of the Imperial and Royal Society of Physicians and Surgeons of Vienna, &c.


### INFLAMMATION AS IT OCCURS IN THE DIFFERENT TISSUES OF THE EYE;

BEING A COMPLEMENT TO THE

*Author's Reports on Inflammation and the Healing Process.*

By T. Wharton Jones, F.R.S.
Lecturer on Anatomy, Physiology, and Pathology at the Charing Cross Hospital, &c.

In Reports contained in the Numbers of this Review for April and July 1844, the nature of inflammation and of its events was analysed. Except for the purpose of occasional illustration, these processes, as they occur in particular structures, were not referred to; what was then said being applicable to inflammation and its events whatever might be the structure affected. But as the phenomena of inflammation are more or less modified according to the structure affected, it is now purposed, in order to complete the history of the subject, to consider inflammation and its events as they present themselves in particular structures.

The particular structures to which reference was made for the purpose of illustrating the general doctrines of inflammation, were certain of those of the eye —an organ generally pointed to by pathologists as that in which inflammation may be most conveniently and perfectly studied. Composed of different tissues, and these for the most part open to direct inspection, the eye, when affected with inflammation, indeed affords an especially favorable opportunity for observing and often for comparing the modifications of the phenomena of inflammation dependent on difference of structure.

On the present occasion, it is purposed still to keep to the eye.

### I. Objective Phenomena of Inflammation of the different Structures of the Eye.

§ 1. In entering on this subject, it is proper to call to mind the following particulars:

1st. *Inflammatory Congestion* is manifested to the naked eye by *preternatural redness*, which, as is well known, is owing to the large quantity of red blood-corpuscles accumulated and stagnant in the small vessels, as before explained.

2d. *Exudation*, when the exuded matter is not directly observed, as it may be in iritis for example, is manifested at first in different ways, according to the structure of the part—e. g. by swelling, thickening, opacity, vesicles, phlyctenulæ, discharge, &c., and subsequently by the new tissues or by the pus, which may be developed from the exuded matter.

3d. *Extravasation of Blood* is manifested either by actual hemorrhage, or by infiltration of the tissue in the form of ecchymosis, &c. The redness of ecchymosis is in general readily distinguished from that of vascular congestion.

### INFLAMMATION OF THE CONJUNCTIVA.

§ 2. There are four principal forms of inflammation, of which the conjunctiva, like other mucous membranes, may be the seat—catarrhal, erysipelatous, pustular or aphthous, and pseudo-membraneous.

#### *Catarrhal inflammation of the conjunctiva.*

§ 3. *Congestion—Redness.* The conjunctiva of the eyelids and of the palpebral sinuses, is deep red. The conjunctiva is also deep red where it is reflected upon the eyeball; but towards the cornea the redness is, at the commencement of the inflammation, gradually shaded off. When, however, the inflammation is fully developed, the redness extends even to the margin of the cornea.

§ 4. The injection of the highly-developed capillary network of the palpebral conjunctiva gives rise to a uniform and intense redness, concealing from view the larger subjacent vessels. Except in a very high degree of inflammation, the injection of the less developed capillary network of the sclerotic conjunctiva does not conceal the larger subjacent vessels. Indeed, what most strikes the observer is the network with large meshes formed by the intercrossing and inosculation of comparatively large and tortuous vessels—the arteries and veins which carry the blood to and from the superficial capillary network.

§ 5. In consequence of the accumulation of blood in its vessels, the conjunctiva is thickened. The papillæ of the palpebral conjunctiva, being for the same reason swollen and erect, the inner surface of the eyelids has a velvety appearance.

§ 6. The conjunctiva covering the caruncula lacrymalis, and forming the semilunar fold, is deep red like the palpebral conjunctiva, and being at the same time thickened by the accumulation of blood in its vessels, both the lacrymal caruncle and semilunar fold appear much enlarged.

§ 7. *Exudation.* At the commencement of the inflammation a serous exudation takes place from the surface of the conjunctiva. By and by, a puro-mucous or purulent discharge, the presence of which is an important character of the inflammation, is established.

§ 8. There is necessarily some exudation into the substance of the conjunctiva itself, producing thickening of it, and enlargement of the papillæ of its palpebral portion.

§ 9. In some cases small phlyctenulæ like pins' heads are observed on the palpebral conjunctiva. These are produced by small collections of exuded matter, raising up the epithelium.

§ 10. The eyelids, besides being somewhat red, may be more or less swollen, from exudation into their cellular tissue. There may also be exudation into the cellular tissue underneath the sclerotic conjunctiva, constituting *chemosis.* Tumefaction of the eyelids and chemosis are analogous in their nature and mode of production to that swelling which takes place in the neighbourhood of any active inflammation.

§ 11. *Extravasation of blood.* Ecchymotic spots sometimes present themselves especially over the sclerotica, in consequence of extravasation of blood into the substance of the sclerotic conjunctiva, or into the cellular tissue underneath. When the inflammation is intense, there may be actual discharge of blood from the surface of the conjunctiva. This more readily takes place from the palpebral conjunctiva.

§ 12. From the description now given of catarrhal inflammation of the conjunctiva, it appears that the matter which is exuded from the inflamed surface is converted into puriform mucus or actual pus, though the surface is not ulcerated. On the other hand, the lymph which may have been exuded into the substance of the membrane is developed into tissue, which is the cause of that thickening of the conjunctiva, and enlargement of its papillæ, which remain after the inflammation has subsided, for a greater or less length of time, or even permanently.

§ 13. *Healing process.* Before a puriform or purulent discharge is established, and before thickening of the conjunctiva and hypertrophy of its papillæ have taken place, consequently, before exudation has been anything more than serous, resolution of the inflammation may occur. In the contrary case, the return to the healthy state proceeds thus: As vascular congestion, or in other words, the redness diminishes, the sero-mucous or puro-mucous discharge becomes less and less, and any accompanying chemosis and swelling of the eyelids subside. It is to be remarked that the vascular congestion of the palpebral conjunctiva disappears less quickly than that of the ocular conjunctiva. The papillæ of the palpebral conjunctiva, moreover, are extremely apt to be left in a state of hypertrophy, constituting the so-called *granulations* of the conjunctiva.

§ 14. This view of the nature of the granulations of the conjunctiva has been called in question by Engel, (Oest. med. Wochenschrift, 1842, No. 9.) He asserts, and Henle agrees with him, that the palpebral conjunctiva does not present a papillary body, and consequently that the so-called granulations cannot be hypertrophy of it. Engel and Henle view "granulations" as new formations resulting from the organization of a plastic product on and in the tissue of the conjunctiva.

§ 15. In regard to this, it is to be observed that the palpebral conjunctiva may present a granular or velvety appearance in the stage of inflammatory congestion, before there can be any question of new formations, owing simply to the enlargement, by injection with blood, of those minute prominences, named papillæ by some, mucous glands by others, with which the tarsal part of the palpebral conjunctiva is beset, and which give rise to its natural fine shagreen appearance: and that there is nothing in Engel's description of fully-developed granulations to show that they are not hypertrophy of these same minute prominences, i. e. enlargement of them by the addition of new organic elements, resulting from the organization of plastic matter, exuded on or in them, together with general thickening of the conjunctiva from the same cause.

*Erysipelatous inflammation of the conjunctiva.*

§ 16. Like inflammation of other mucous membranes, inflammation of the conjunctiva sometimes presents itself with characters between inflammation and œdema. There is considerable infiltration of serum into the substance of the membrane itself as well as into the subjacent cellular tissue. The disease is known by the name of *erysipelatous ophthalmia.*

§ 17. The most remarkable appearance in this ophthalmia is the watery exudation under the sclerotic conjunctiva, whereby the latter is raised up in folds which protrude like vesicles between the eyelids. The conjunctiva is of a light red colour, inclining to yellow, and presents here and there spots of ecchymosis, but individual vessels are not readily discernible. The mucous secretion of the conjunctiva is somewhat increased in quantity.

*Pustular or aphthous inflammation of the conjunctiva.*

§ 18. *Congestion—Redness.* The sclerotic conjunctiva presents one or several small scarlet spots, produced by the convergence of vessels. At the spots mentioned the vessels are large and evident, but as they recede from the spots, they cease to be distinguished by the naked eye; hence the vessels of the red spots appear as if isolated from all connexion with any other. By and by, however, the continuity of some of the vessels of the red spots, with those of the rest of the conjunctiva, comes to be distinctly seen.

§ 19. *Exudation.* The exuded matter first distinctly manifests itself whilst in process of metamorphosis into pus as a small yellow flake in the centre of the vascular spots.

§ 20. In consequence of the little density and cohesion of the epithelium of the conjunctiva scleroticæ, it does not, like the epidermis of the skin, retain the exuded matter, but gives way, so that a proper pustule is not formed, but an aphthous spot or small abrasion covered with the exuded matter, becomes pus or puriform, and fragments of epithelium.

§ 21. Pustules often present themselves close to the margin of the cornea. In this case the vessels do not converge from all points to the pustules, but come from the side of the sclerotic conjunctiva only, as indeed was to be expected, seeing that none could come from the cornea on account of its being non-vascular. The thick epithelium of the cornea is at the part opaque and slightly raised by the exuded matter which, with the softened epithelium of the sclerotic conjunctiva of the spot affected, forms, as in the preceding case, the small yellow flake. This form of pustule thus presents a character intermediate between the phlyctenula or pustule of the cornea and the aphthous spot of the sclerotic conjunctiva above described.

§ 22. *Extravasation of blood.* Besides the red spots from vascular congestion just described, there may be patches of ecchymosis.

§ 23. *Healing process.* Pustules or aphthæ of the conjunctiva may run into ulceration, but in general the spots of vascular congestion disappear, and the abrasion produced by the separation of the epithelium, quickly heals, the spot becoming covered with a new epithelium, whilst the coating of puriform matter and fragments of old epithelium is thrown off.

*Inflammation of the conjunctiva, with pseudo-membraneous exudation.*

§ 24. In inflammation of mucous membranes it is well known that the matter exuded on the surface is sometimes found in the form of false membranes. This matter, however, does not, like that exuded by serous membranes, become organized into tissue; such false membranes are usually separated and thrown off.

§ 25. An approach, perhaps, to pseudo-membraneous exudation, occurring in inflammation of the conjunctiva, presents itself in the yellow membraniform flakes, commonly considered as flakes of puro-mucus, which are thrown off in the purulent ophthalmiæ. In some cases of phlebitic ophthalmia there are layers of exuded lymph on the conjunctiva.

§ 26. The coagulation and exfoliation of the epithelium, especially of the cornea, in consequence of chemical injuries, have been mistaken for pseudo-membraneous formation.

*Mortification and ulceration of the conjunctiva.*

§ 27. Mortification of the conjunctiva, as a consequence of inflammation, does not appear to have been met with, but sloughing of parts of the conjunctiva, in consequence of chemical injury, sometimes occurs. Ulceration, except from a specific cause, seldom takes place.

*Healing of wounds of the conjunctiva.*

§ 28. Wounds of the conjunctiva gape much but readily heal. The conjunctiva becomes injected at the edge of the wound, and lymph is exuded, which becomes organized in the manner already explained, according as the union is by the first or second intention, one or the other event being in general determined by the apposition or non-apposition of the edges of the wound, as in the skin.

§ 29. The palpebral and ocular surfaces of the conjunctiva have no tendency to form adhesions even while kept in close apposition, unless previously made raw. When abrasion of the surfaces has been produced, especially by burns and escharotics, there is then great tendency to the formation of adhesions.

INFLAMMATION OF THE SCLEROTICA.

§ 30. *Difference between conjunctival injection and sclerotic injection.* In conjunctival inflammation it has been seen that the vessels of the sclerotic conjunctiva are large, somewhat tortuous, and arranged in a reticular manner; that the colour is scarlet, or brick red, and that it may be deeper towards the orbit, but more or less shaded off towards the cornea. In sclerotic injection the redness is in the form of a pink or lake coloured zone, encircling the cornea; the injected vessels being very minute, and disposed in straight radiating lines, as if from the margin of the cornea, where the tint is deeper, whilst it is shaded off, and disappears towards the orbit—the converse of what occurs in the injection attending conjunctival inflammation.

§ 31. The seat of the injected vessels, whether in the sclerotic conjunctiva or in or on the sclerotic itself, is easily proved, supposing any doubt exist, by making the conjunctiva slide on the sclerotica, when the vessels, if seated in the conjunctiva, will be observed to move along with it, whereas, if seated in the sclerotica, or closely applied to its surface, they will remain stationary. When both conjunctiva and sclerotica are injected at the same time, the pink hair-like vessels of the sclerotica are seen stationary through the larger meshes of the sliding conjunctiva. But when the conjunctiva is very much injected, the state of the sclerotica cannot be seen.

§ 32. If the vascular congestion be alone taken as inflammation, then it must be said that the part of the sclerotica visible during life through the conjunctiva, is often inflamed, but if exudation, and the changes which the exuded matter undergoes, be rather assumed to be indicative of inflammation, then it must be admitted that the sclerotica is comparatively rarely the seat of inflammation.

§ 33. Fibrous tissues in general do not appear to be more frequently the seat of the effects of inflammation than the sclerotica, but are they not as frequently the seat of vascular congestion? Is rheumatism anything more in most cases than vascular congestion in fibrous tissues, with perhaps serous exudation in neighbouring parts? What is called rheumatic ophthalmia appears to be at least nothing more than inflammatory congestion of the sclerotica, usually, with more or less implication of the iris.

§ 34. Rheumatism, or inflammatory congestion in fibrous structures, may at last lead to exudation of lymph either into the substance or on the surface of the part affected,—in the one case giving rise to the thickening and induration of the fibrous structures, in the other, to effusions into the joints or adhesions, such as are met with in pericarditis. By repeated congestions, the sclerotica is indeed left in a somewhat altered state, but it is the cornea or iris which is principally the seat of exudation of lymph and the changes consequent on it, as the joints are in articular rheumatism.

§ 35. The most marked example perhaps of the tissue of the sclerotica becoming the seat of changes from inflammation occurs in sclerotico-choroiditis. The first change is a thickened and fleshy appearance of the sclerotica, but its texture becoming at the same softened, it by and by yields to the distension from within the eye, protrudes and becomes attenuated, the dark colour of the choroid shining through, (*sclerotic staphyloma.*) In some cases, however, instead of becoming attenuated, the affected part of the sclerotica actually becomes thickened, of a dense white pearly appearance.

### INFLAMMATION OF THE CORNEA.

§ 36. *Congestion—Redness.* On account of the non-vascularity of the cornea, there is at first no redness of it from vascular congestion. Congestion is not, however, wanting, but is seated in the adjoining conjunctiva and sclerotica, as already explained. (Report on Inflammation in this Review for April, 1844.)

§ 37. *Exudation.* That the cornea is the seat of exudation is manifested by opacities of various kinds, phlyctenulæ, and abscesses. When new vessels are developed in the exuded matter, the cornea then becomes the seat of more or less redness. This, however, is to be distinguished from that which may result from effused blood. When effusion of blood occurs, it appears usually in a patch near the edge of the cornea.

§ 38. One or other of the three principal layers of the cornea may be more particularly the seat of the exuded matter; hence there are distinguished inflammation of the proper substance of the cornea, inflammation of the conjunctiva corneæ, and inflammation of the membrane of Descemet.

### *Inflammation of the proper substance of the cornea.*

§ 39. In inflammation of the proper substance of the cornea, the vascular congestion is seated in the sclerotica in the form of the circumcorneal sclerotic zone, but sometimes the redness is very slightly marked. There is generally also congestion of the circumcorneal network of the conjunctiva.

§ 40. The exuded matter is deposited either in the interstices of the tissue or on its surface, raising the epithelium up in the form of a phlyctenula, or even a blister.

§ 41. The exudation into the interstices of the proper substance of the cornea, may produce map-like patches of dimness and nothing more. Or the exudation being in greater quantity, a general grayish or yellowish white opacity results, denser at some points than others, and intermixed with red from the presence of new vessels. In this case, the cornea presents a peculiar opalescent appearance. In certain cases there is less exudation and development of vessels; the cornea still retains a degree of transparency, but is of a dirty yellowish green colour, and rough like ground glass, owing to minute vesicles on its surface, or minute points of ulceration, resulting from the bursting of the vesicles. There is softening of the cornea in all these cases.

§ 42. When exudation into the proper substance of the cornea, or under the epithelium, takes place rapidly and copiously, the exuded matter is generally formed into pus or puriform matter, and the result is an abscess or a pustule. In such cases the inflammation is more of an acute character than in the preceding. There is more vascular congestion in the conjunctiva and sclerotica, so much so that the cases in question are commonly viewed as examples of corneitis supervening on inflammation of the conjunctiva and sclerotica, while the preceding cases are, on account of the slight appearance of congestion in the conjunctiva and sclerotica, viewed as examples of primary corneitis. But from what has been above said of inflammation of the cornea, there is no *primary corneitis* in the sense here implied, i. e. with vascular congestion first in the cornea.

§ 43. The depositions of yellow matter which occur in the interstices of the cornea at its lower part, and which, on account of their presenting the form of the lunular spot at the root of the nails, are called *unguis* or *onyx*, and which are in general rapidly absorbed as the attendant inflammation is subdued, have less of the character of abscesses than the circumscribed collections of matter which form in the centre of the cornea. These latter make their appearance as a densely opaque spot, first white then yellow, around which the rest of the cornea is more or less opaque from exuded lymph, in which there may be new vessels, as in the walls of abscesses elsewhere.

§ 44. Most frequently the exuded matter is deposited on the surface of the proper substance of the cornea, raising up the epithelium in the form of a phlyctenula or blister. The epithelium of the cornea being denser, thicker, and more coherent than that of the sclerotic conjunctiva, confines the matter which is exuded, in much the same way that the epidermis of the skin does. The matter being at first a transparent fluid, there is a phlyctenula; subsequently becoming puriform or purulent, there is a pustule. Often the process does not proceed so far as the formation of a pustule.

§ 45. A phlyctenula or pustule of the cornea having burst, a small ulcer covered with puro-lymph is left, which may be compared to the aphthous spot on the sclerotic conjunctiva. A fasciculus of new vessels, extending to this ulcer from the circumcorneal conjunctival network, may make its appearance.

§ 46. *Healing process.* When the congestion around the cornea subsides, the matter exuded into its substance may gradually be absorbed. And this even when development of it has gone on to the formation of new vessels, though tardily, for the more the exuded matter has been developed, the less readily does it dissolve and become fitted for absorption. The new vessels first disappear, leaving a grayish white opacity, which clears away from the circumference towards the centre of the cornea, where often more or less opacity remains.

§ 47. A pustule on the surface of the cornea, or an abscess in its proper substance, may disappear by absorption of its contents, leaving, however, more or less opacity: but these collections of matter usually burst, and leave a sore, which may either commence to heal by granulation or run into ulceration.

### *Inflammation of the conjunctiva corneæ.*

§ 48. This usually accompanies acute inflammation of the proper substance of the cornea, or is an extension of inflammation of the sclerotic conjunctiva.

§ 49. In consequence of the exudation, the conjunctiva corneæ becomes at some point opaque and thickened, and here new vessels are soon formed, which, connecting themselves with the circumcorneal conjunctival network—which at the place is in a state of congestion—appear as a mere extension of a fasciculus of vessels from it. The opacity and vascularity may gradually spread across the cornea.

§ 50. In some cases of what may be called inflammation of the conjunctiva corneæ, there are fewer new vessels and less opacity, but there is superficial spreading ulceration. In certain cases the cornea presents here and there on its surface vascular fungous granulations.

§ 51. The changes which the conjunctiva corneæ undergoes in inflammation, the thickening and vascularity, are very apt to remain in the forms of pannus, vascular cornea, &c.; but often they disappear entirely, and the cornea resumes its natural appearance.

*Inflammation of the membrane of Descemet.*

§ 52. In this inflammation, the vascular congestion is in the sclerotic zone. The exuded matter is deposited between the proper substance of the cornea and the membrane, and generally presents itself in the form of scattered punctiform opacities. Here also new vessels, when formed, make their appearance.

§ 53. As the inflammatory congestion subsides, the exuded matter is removed by absorption.

*Ulceration of the cornea.*

§ 54. The cornea is extremely prone to ulceration. The ulceration may be limited to a mere abrasion or exfoliation of the epithelium, or it may affect the proper substance of the cornea also. The membrane of Descemet does not appear to be liable to ulceration; but when exposed and deprived of support by penetrating ulceration of the proper substance of the cornea, it bursts.

§ 55. *Abrasion of the epithelium* presents itself either in that form, in which its surface looks like ground glass, or in a form like what is presented after death, when the epithelium begins to soften, and portions of it are detached by wiping the surface. The first form occurs in inflammation of the proper substance of the cornea. The second is rather a result of inflammation of the conjunctiva corneæ; there is superficial vascularity, and the abrasion, like ulceration, has a great tendency to spread; but while it spreads on one side, cicatrization may be seen taking place on another. The cicatrization gives rise to slight opacity.

§ 56. *Ulceration of the proper substance of the cornea.* This generally commences by the bursting of an abscess or phlyctenula. Both the bottom and edges of the ulcer may be clear, and the cornea around scarcely, if at all, nebulous. In other cases the bottom of the ulcer is filled with a grayish sloughy-looking matter, which is thrown off to be succeeded by the same thing, whilst the ulcer goes on increasing in depth, and may at last completely perforate the cornea.

*Mortification of the cornea.*

§ 57. The complete death of the cornea, and the separation of it in the form of a well-marked leathery slough, is of rare occurrence. The destruction of the cornea, which is so common in the purulent ophthalmiæ, takes place in a different manner.

§ 58. The cornea, overlapped all round its margin by the chemosed conjunctiva, may be observed to continue for some time unaffected; but within a short interval it will be found to have become quite opaque and softened. To this succeeds the process of destruction, which consists in that form of mortification, with small sloughs, which constitutes ulceration. The destruction may involve the whole cornea in its whole thickness, or a part only in its whole thickness; or it may involve a superficial portion only, and this of a greater or less size.

§ 59. The immediate cause of all this mischief appears to be the infiltration of the substance of the cornea with exuded matter, and the mechanical pressure exerted by the chemosed conjunctiva, whereby the nutritive movements are more or less completely arrested.*

* Though the cornea may become vascular in the course of inflammation from the development of new vessels, this does not apply to all non-vascular parts, to cartilage for example. The vessels which have been described as making their appearance in cartilage in the course of inflammation of joints do not properly speaking exist in cartilage, but in a new tissue which has supplanted the cartilage; this having been changed and destroyed in consequence of the inflammatory congestion in the parts from which it derives the materials for its nourishment. This view, which the author of this paper considers the correct one, is that given by Rokitansky and agreed to by Henle.

*Healing of wounds and ulcers of the cornea.*

§ 60. *Healing by the first intention.* A simple incision of the cornea readily heals. From the vessels of the conjunctiva and sclerotica which are congested on that side of the cornea next the wound, lymph is exuded into the cornea at the seat of the wound, producing opacity to a greater or less extent around, and of more or less intensity. The cut edges are agglutinated by the exuded lymph, and by its organization continuity of structure is restored. What of exuded matter remains in the substance of the cornea around, producing opacity, is gradually absorbed, and the cornea clears in proportion as the injection of the conjunctival and sclerotic vessels subsides; a small speck perhaps, the cicatrice, merely remaining. No new vessels may have been formed in the cornea.

§ 61. *Healing by the second intention.* Loss of substance of the cornea, whether produced by ulceration or otherwise, is restored by granulation. The granulations may be non-vascular, or they may be vascular, from new vessels which have been developed in the exuded matter, and which have formed a connexion with those of the neighbouring conjunctiva and sclerotica. These new vessels generally disappear when the process of granulation is completed, and preparatory to cicatrization. Thus, when an ulcer has filled up by vascular granulations, one vessel after another disappears, until all are gone, leaving an opaque streak where their course in the cornea had been.

§ 62. At first the sore may be swollen, and more or less nebulous at the edges, and discharge a tough, yellow, puro-lymphy matter, which sometimes adheres to it, and hangs down from it in flakes. But when the ulcer begins to heal, its edges become decidedly gray and opaque, and in proportion as it becomes filled with granulations, the quantity of puro-lymphy matter discharged from it becomes less, until none at all is formed. At last cicatrization takes place, and the surrounding nebulosity diminishes, until it disappears altogether.

§ 63. The cicatrice is either a permanently opaque spot (*leucoma*) or it is a clear facet, presenting the appearance as if a small piece had been sliced from off the convex surface of the cornea.

### INFLAMMATION OF THE IRIS.

§ 64. *Congestion—Change of colour of the iris.* In consequence of the coloration of the iris, it does not, like the conjunctiva for example, when inflamed, appear red, but of a colour which is a compound of its own natural colour and that of the stagnant blood. Thus a blue iris becomes green, a brown iris reddish brown. The brilliancy of the iris is at the same time impaired or lost. Subsequent changes in the colour of the iris are owing to exuded matter and to changes in the pigment.

§ 65. The injected vessels are individually not very evident. Such may sometimes be seen, however, and are to be distinguished from those of new formation which make their appearance at a later stage of the inflammation.

§ 66. *External redness of the eyeball.* The circumcorneal sclerotic red zone is well marked. The conjunctiva may be little or very much injected—so much sometimes as to hide the sclerotic injection.

§ 67. *Exudation.* The aqueous humour is at first somewhat increased in quantity by exudation of serum. Exudation of lymph afterwards takes place. Lymph may be exuded on the surfaces or into the substance of the iris. The exudation from the anterior surface and from the pupillary margin may be directly seen. Most commonly the exudation appears first at the pupillary margin. On the anterior surface the lymph presents itself in drops, and fine flakes of it may often be seen in the aqueous humour, rendering it turbid.

§ 68. The lymph exuded at the pupillary margin soon becomes consolidated and organized, forming bands of adhesion between the margin of the pupil and the capsule of the lens—distorting the pupil, and sometimes also producing closure of it to a point.

§ 69. The mode in which closure of the pupil takes place appears to be this: the pupil having been in a state of contraction when the lymph was exuded, this lymph, in consolidating, contracts and draws together more closely the margins of the pupil from which it has been exuded, and to which it is adherent.

§ 70. New vessels may make their appearance in the lymph exuded on the anterior surface of the iris, and also in that filling up the pupil.

§ 71. The lymph poured out at the pupillary circle, or at the margin of the pupil, forming bands of adhesion, becomes of a brown or yellow colour from the development of pigment in it.

§ 72. Though no distinct serous membrane can be demonstrated on the anterior surface of the iris, it thus, like the surface of organs covered with serous membranes when inflamed, pours out lymph which gives rise to adhesions ; but what is peculiar is, that the adhesion which takes place is between the iris and capsule of the lens only, rarely between the iris and the cornea.

§ 73. *Parenchymatous inflammation of the iris* may be looked upon merely as a more intense degree of inflammation, in which, to exudation on the surface of the iris, there is added exudation into its substance, and that quickly and in large quantity.

§ 74. There is, in acute parenchymatous inflammation of the iris, greater vascular congestion both of the conjunctiva and sclerotica, together with congestion of the choroid, as may be inferred from the accompanying photopsia during life, and from its having been found on dissection after death that there was lymph on the inner surface of the choroid.

§ 75. Exudation into the substance of the iris takes place principally at the pupillary circle, or at the ciliary part, less frequently in the middle. It is manifested by the iris losing much of its natural appearance of structure, and becoming swollen, with its pupillary edge retracted, and its middle bolstered forward.

§ 76. Abscess is apt to form in such cases when acute. It appears as a small reddish yellow tubercle on the surface of the iris, generally near its pupillary edge, which bursting into the anterior chamber, gives rise to a small hypopyon. When the abscess is quite at the ciliary margin, it may evacuate itself externally through the sclerotica, close to the place of its junction with the cornea.

§ 77. *Effusion of blood.* Hemorrhagic exudation also occurs in inflammation of the iris. Besides the small quantity of blood often effused, forming a patch of greater or less size on the anterior surface of the iris, and tinging the exuded lymph, blood is sometimes poured out in such quantity as to fill the aqueous chambers.

§ 78. *Healing process.* As the congestion in iritis subsides, the progress of the absorption of the exuded matter is beautifully seen. Matter which has been recently exuded rapidly disappears ; that which has become already organized into adhesions, having to undergo solution by a retrogressive metamorphosis, in order to be fitted for absorption, disappears more slowly ; and in many cases the organization is so complete, that no such process of removal takes place. Thickening and change of structure of the iris from exudation into its substance, together with contraction of the pupil and obstruction of it with lymph, are very often permanent.

§ 79. Effused blood is in general readily absorbed, but after repeated effusions some remains unabsorbed, in the form of brown or black masses and patches, at the bottom of the anterior chamber and on the surface of the iris.

§ 80. *Inflammation of the lining membrane of the posterior chamber or uveitis.* Lymph may be exuded in large quantity into the posterior chamber, on the posterior surface of the iris, and on the anterior wall of the capsule of the lens. The source of this exudation is probably the vessels of the ciliary processes. See further on this head, infra § 89.

### INFLAMMATION OF THE CHOROID AND RETINA.

§ 81. The anatomical characters of the inflammations of these structures cannot be directly observed during life ; even the external redness of congestion cannot be seen, as, different from the structures hitherto considered, the blood-vessels of those under consideration enter the eyeball at its posterior part. The cases moreover in which there has been opportunity for examination of the eye after death, have in general been such as presented the effects of past inflammation rather than the manifestations of inflammation in progress. But from analogy with what has been seen in inflammation of the other structures of the eye, and from what has been observed in the *post-mortem* examinations referred to, the anatomical characters of inflammation of the choroid and retina may be inferred with some degree of probability.

*Inflammation of the choroid.*

§ 82. *Congestion.* The capillary network on the inner surface of the choroid will be more or less injected, and the larger vessels proceeding to or from it, and which are principally seated on its exterior, will be enlarged, so that the choroid will be much increased in thickness, and will press inwards upon the retina and outwards upon the sclerotica. There will be much redness at the posterior part of the sclerotica, from the injection of the choroidal or short ciliary vessels.

§ 83. *Exudation.* Exuded matter will be deposited between the choroid and membrane of the pigment, raising and breaking up the latter, together with the delicate stratum bacillosum of the retina, or producing adhesions between the choroid and retina, with alteration of their texture. That something like what is here supposed does really occur is evidenced by what has been found in the few cases in which there has been opportunity for examining the eyes after death. Exudation may also take place between the choroid and sclerotica.

§ 84. If there should be such intense congestion as is presented by the conjunctiva in purulent ophthalmia, a large quantity of matter will be exuded, and suppuration will be the result, with breaking up and disorganization of the whole interior of the eye. But before this, the other internal structures will have become implicated. The case will thus now be one of general ophthalmitis or ocular phlegmon.

*Inflammation of the retina.*

§ 85. *Congestion. Redness* of the inner surface of the retina from injection of the ramifications of the central artery of the retina.

§ 86. *Exudation.* Exuded matter between the retina and vitreous body, and also into the substance of the vitreous body, and into that of the retina itself. The effect of this is degeneration of the retina, the vitreous body, and subsequently of the posterior capsule of the lens.

INFLAMMATION OF THE LENSES OF THE EYE.

§ 87. These bodies being, like the cornea, non-vascular in the fully developed state, inflammation of them consists at first merely in exudation into or on them; the vascular congestion having its seat in adjacent parts.

*Inflammation of the crystalline body.*

§ 88. Inflammation of the crystalline body is first evidenced by opacity of the capsule, resulting from exudation into or on it. In the exuded matter new vessels may be developed. But where is the seat of the primary congestion? This appears to be different according as it is the anterior or the posterior wall of the capsule which is affected.

§ 89. In uveitis, § 80, the anterior wall of the capsule has often exuded matter deposited on it, in which new vessels are sometimes developed. This is the kind of case described as inflammation of the anterior wall of the capsule.

§ 90. Whilst inflammation of the anterior wall of the capsule belongs to the head of inflammation of the anterior segment of the eye, what has been viewed as inflammation of the posterior wall of the capsule comes under the head of inflammation of the posterior segment.

§ 91. The vessels described by Walther in the posterior wall of the capsule, radiating from the centre, as indicative of inflammation of the posterior wall of the crystalline, cannot be, as has been supposed, the enlarged ramifications of the central artery of the vitreous humour, for these have become in the developed eye entirely obliterated. When vessels exist, they must be new formations, developed in exuded lymph. The author of this Paper has not seen any case in which red vessels were actually visible; but he has seen radiating streaks of opacity in the situation of the posterior wall of the capsule somewhat similar in arrangement to that of the vessels represented in Professor Walther's figure of inflammation of the posterior wall of the capsule.

§ 92. When the capsule of the lens is affected, as above described, the lens itself becomes more or less altered in consequence. It becomes opaque, dissolved, or is even the seat of suppuration. Vessels, it is alleged, have been observed shooting into it from the inflamed capsule.

§ 93. *Healing process in the crystalline body.* Wounds of the crystalline body, it is well known, are in the human eye very generally followed by opacity of the lens. In experiments on brutes this result has occurred in some cases only. As to the capsule, it becomes opaque in the seat of the wound, from exudation depending on the inflammatory congestion which has been occasioned in neighbouring structures by the wound, of which that of the crystalline is necessarily a part only. The wound of the capsule may thus unite. If the wound of the capsule is large, and does not unite, the opaque lens, it is known, dissolves and disappears.

§ 94. *Regeneration of the lens.* Pauli, Lowenhardt and Textor have repeated the experiments on regeneration of the lens in animals with success. Textor communicates some new cases of regeneration of the lens in man after operations for cataract. The proof that the newly-formed substance possesses the same intimate structure as the lens has at last been supplied by Valentin's microscopical investigation of the subject.*

*Inflammation of the vitreous body.*

§ 95. This does not appear to take place without the posterior wall of the capsule of the lens also becoming affected. Again, though in the fully formed eye there may still exist hyaloid vessels, as described by Arnold and Van der Kolk, the congestion on which inflammatory changes of the vitreous body principally depend, is congestion of the vascular layer of the retina. § 86.

§ 96. The objective phenomena of inflammation, and its events, having thus been considered, as they occur in the different textures of the eye, those especially open to inspection during life, a general survey may now be taken of the modifications which those phenomena present, according to the structure affected.

§ 97. *Phenomena of congestion.* It has been seen that the more vascular conjunctiva, when inflamed, is redder than the less vascular sclerotica; and that the non-vascular cornea is not red at all, but that the congestion, and consequently the redness attending inflammation of it, are seated in adjacent parts. It has been seen, however, that redness of the cornea itself may be subsequently superadded by the development of new vessels in it. It has been seen, lastly, that in the coloured iris, the congestion is not manifested by redness, but by a colour a compound of the yellowish redness of a thin stratum of blood and the natural colour of the inflamed structure.

§ 98. *Phenomena of exudation.* It has been seen that exudation takes place more copiously from the conjunctiva and iris than from the less vascular sclerotica, and that, cæteris paribus, the exudation is in proportion to the degree of inflammatory congestion. It has been seen that the exuded matter is for the most part poured out from the surfaces of the conjunctiva and iris, and that there is little

* Henle's Bericht, p. 279.

swelling and thickening, manifesting interstitial exudation, in comparison with the whole quantity of matter exuded; whereas in parenchymatous structures, the exuded matter being received into their interstices, exudation is manifested by more or less considerable swelling. It has been seen that exudation gives rise to phlyctenulæ and pustules on the surface of the cornea, but not so readily on that of the conjunctiva, in consequence of the difference in the thickness of the epithelium investing the two surfaces. Exudation, it has been seen, may take place into the cellular tissue underneath the conjunctiva in inflammation of that membrane, in which case there is the swelling called chemosis. Lastly it has been seen, that in congestion of the sclerotica there is comparatively little disposition to exudation, and that when it does take place, it is often rather into the neighbouring cornea than into the substance of the sclerotica itself—a peculiarity which seems to hold in the case of other fibrous structures, those around joints, for example, in rheumatic gout.

§ 99. *Phenomena of the extravasation of blood.* It has been seen that from the surface of the conjunctiva, when it is the seat of intense inflammatory congestion, slight hemorrhage readily occurs; but that in less intense inflammation, extravasation of blood occurs in the form of large patches of ecchymosis into the loose cellular tissue underneath the sclerotic conjunctiva. It has been seen that effusion of blood may take place from the surface of the inflamed iris, analogous to the hemorrhagic exudations of inflamed serous membranes—and that extravasation may also occur into its substance. But in this latter case, as also in the case of extravasation into the substance of the cornea, the spots of ecchymosis are small in comparison with those which present themselves in the looser subconjunctival tissue. The readiness with which bleeding takes place from the surface of the conjunctiva, when the seat of intense congestion, is explicable by the exposure to foreign contact of its delicate superficial capillary network in a state of great distension.

§ 100. *Phenomena of the events of inflammation.* A modification of the phenomena of the events of inflammation might *à priori* be presumed to occur in different structures, in consequence of that physiological difference which determines the mode of assimilation peculiar to each structure. That such a modification holds to a certain extent only, and is readily broken through by modifying influences, is shown by the formation of pus in very different structures, and by the circumstance that a kind of cellular tissue and blood-vessels are the new structures most commonly regenerated, whatever the original structure may be.

§ 101. An influence which manifestly modifies the manner in which the exuded matter is disposed of consists in the exposure or non-exposure of it to the contact of foreign bodies, including the external air. In the inflammations above passed in review it was seen, that the matter exuded on the surface of the conjunctiva in contact with the external air, tends to be converted into pus or puriform matter, whilst that exuded on the surface of the iris out of contact with air, is more disposed to be converted into tissue, forming bands of adhesion.

§ 102. The mode in which exposure to the contact of foreign bodies operates in determining suppuration is probably by their irritation keeping up the congestion, and thus causing exudation in large quantity and of a certain quality. In the cases in which the exuded matter is converted into pus, though not in contact with foreign bodies, the exuded matter has been deposited in large quantity, in consequence of the greatness of the congestion from other causes.

§ 103. In the cornea there may be observed what will perhaps be admitted as an exemplification of the influence of comparative quantity of exuded matter in the disposal of it. When exudation takes place slowly and in small quantity, it is developed into tissues; but when exudation takes place rapidly and in large quantity, suppuration results.

§ 104. The disposition of the iris to form adhesions with the capsule of the lens, as in the case of serous surfaces, presents a remarkable contrast to the indisposition which, in common with other mucous surfaces, those of the inflamed conjunctiva have to adhere, even when in close apposition, except when abraded, and therefore no longer mucous surfaces. This appears to point to some peculiarity in the matter, considered as a blastema, exuded from mucous surfaces. Sometimes, indeed, the matter exuded on mucous surfaces presents itself in the form of pseudo-membranes (§ 24); these, however, do not become organized, like the pseudo-membranes of serous surfaces, but are eventually separated and thrown off like dead parts.

§ 105. In regard to the formation of adhesions between the iris and capsule of the lens (*synechia posterior*) it has been contended (Dr. G. Hoering, Ueber den Sitz und die Natur des grauen Staars, Heilbron, 1844,) that the condition for their formation is not exudation of plastic lymph from an inflamed iris alone, but that the capsule as well as the iris must be in a state of inflammation at the same time.

§ 106. However this may be as regards serous membranes generally, it is to be observed of the case under notice, that seeing inflammation of the anterior wall of the capsule of the lens consists at first merely in exudation into or on it, the exuded matter having its source in congestion of neighbouring parts, there can scarcely be any difference whether the lymph is exuded from the pupillary margin of the iris, or whether it is exuded from the same source as it is in those cases which are considered as properly coming under the head of anterior capsulitis, § 89. And it must be admitted, that synechia posterior occurs in cases in which it would be rather too much to say, that in addition to the iritis, there was anterior capsulitis also.

§ 107. On the other hand, there is great indisposition to the formation of synechia anterior, even when the corresponding surfaces of both iris and cornea are inflamed and in contact, except when there is abrasion of the corneal surface.

§ 108. *Mortification and ulceration.* Of the different structures of the eye above considered, it has been seen that the cornea is that most prone to mortification and ulceration, though perhaps the changes which sometimes take place in the lens and vitreous body might properly be referred to this head.

### II. Subjective Phenomena of Inflammation of the different Structures of the Eye.

§ 109. In entering on this part of the subject, it is, in the first place, necessary to distinguish between the morbid sensations depending on perversion of common sensibility and those depending on perversion of special sensibility.

§ 110. *Morbid sensations depending on perversion of common sensibility.*

1st. *Heat.* The subjective increase of heat, or the patient's sensation of increased heat, depends partly on the objective increase of heat, or that actual increase of heat ascertainable by the thermometer, and partly on an increased susceptibility of the sensitive nerves of the part affected.

Though the more vascular and nervous a part is, the greater in general is the increase of heat, both objective and subjective, in it when inflamed, it is at the same time to be remembered, that there is often greater general reaction and greater inflammatory fever, and consequently greater production of heat in the system in general, from inflammation of parts but poorly supplied with vessels and nerves.

2d. *Pain.* This is owing to the irritation of the nerves of common sensation of the part, already in a state of excitement, produced by the pressure exerted by the congested vessels and the exuded matter. Other parts than that which is the immediate seat of the inflammation may be sympathetically affected with pain.

The pain accompanying inflammation of parts of loose texture, and which have scope to swell, is in general not severe; but in parts of dense texture, or which are so situated that they cannot yield to distension by the accumulated blood or exuded matter, the pain is very severe. When there is much throbbing in an inflamed part, (the nature of which was explained in a former paper,) the attending pain is aggravated at each pulsation, in consequence of the increase of pressure occasioned at the time by the afflux of blood.

§ 111. *Morbid sensations depending on perversion of special sensibility.*

When nervous structures endowed with special sensation are irritated, the sensation produced is not pain, but various modifications of the sensation peculiar to the structure, and this whatever be the irritating agent. Thus when the retina is in a morbidly sensible state, irritation of it by light gives rise to a dazzling glare, which is so distressing, that the patient seeks to protect the eye against light. This constitutes intolerance of light, or photophobia. But even in the dark the same dazzling glare, or various kinds of luminous spectra, may be produced by pressure, &c., and that in a degree more or less distressing, according to the morbid sensibility of the retina, and the intensity of the pressure or other irritating cause. This constitutes photopsia.

Of course actual pain may be experienced at the same time, in consequence of parts endowed with common sensibility being at the same time subjected to the irritating cause which determines the morbid sensations in the retina.

*Morbid sensations depending on perversion of common sensibility, accompanying inflammation of the different tissues.*

§ 112. *Conjunctiva.* Like other mucous membranes close to the natural apertures of the body, the conjunctiva is endowed with a high degree of common sensibility; but being loose in texture the pain which attends inflammation of it is not very severe. There is, however, considerable heat.

§ 113. The most characteristic pain is like that produced by a foreign body in the eye—a sensation which attends inflammation of other mucous membranes near the surface of the body. The sensation as if a foreign body were in the eye is owing to enlargement of the vessels on the one hand, and to increased sensibility of the conjunctiva on the other.

§ 114. Attendant on inflammation of the conjunctiva there are also itchiness and smarting at the edges of the eyelids, with occasional stitches of pain shooting from them.

§ 115. *Sclerotica.* Very severe pain of a rheumatic character around the orbit, in the temples, &c., is, it is well known, a characteristic attendant on sclerotic inflammation or congestion, owing either to accompanying congestion in the parts mentioned, or to nervous irradiation.

§ 116. *Cornea.* The sensation in the cornea itself is one of pressure. But as inflammation of the cornea is attended with injection of the conjunctiva and sclerotica, there may be also the sensation of a foreign body in the eye peculiar to the former, and the rheumatic pain peculiar to the latter.

§ 117. *Iris.* When the iris is inflamed, there is necessarily more or less sclerotic congestion, hence the sclerotic rheumatic pain which so often accompanies iritis. As to the pain within the eyeball itself, it may be accounted for as much perhaps by the distension to which the exterior tunics are subjected by the increased accumulation of blood and fluids in the interior of the eye, as by supposing it to be seated in the iris, which indeed does not appear to possess much sensibility —its numerous nerves appear to be for the most part motor.

§ 118. *Choroid.* The choroid itself does not appear to be endowed with any sensibility. The pain which attends inflammation of it appears to be owing to the distension of the eyeball, and to attending congestion of other parts.

§ 119. *Retina.* The morbid sensations depending on perversion of common sensibility, which may attend inflammation of the retina, have not as has been said their seat in the retina, but are merely owing to distension of the eyeball, and accompanying congestion of other parts.

*Morbid sensations depending on perversion of the special sensibility of the retina.*

§ 120. The appearance of a gauze or mist, or "a skin with veins in it" appears to be the proper subjective effect of the congestion and exudation in inflammation of the vascular layer of the retina.

§ 121. The other special morbid sensations, photopsia, and a morbid sensibility to common impressions, photophobia, occur rather as accompaniments of inflammation of other structures of the eye than of the retina itself. Thus the morbid sensibility of the retina on which intolerance of light depends is an accompaniment of those acute inflammations in which the cornea is especially involved. Luminous spectra again appear to be especially occasioned in inflammation of the choroid, by the pressure arising from the congestion and exudation. This is illustrated by the well-known effect of pressure with the point of the finger on the exterior of the eyeball.

---

